# *N*-Glycolylneuraminic Acid (Neu5Gc) Null Large Animals by Targeting the CMP-Neu5Gc Hydroxylase (CMAH)

**DOI:** 10.3389/fimmu.2019.02396

**Published:** 2019-10-15

**Authors:** Andrea Perota, Cesare Galli

**Affiliations:** ^1^Laboratory of Reproductive Technologies, Avantea, Cremona, Italy; ^2^Fondazione Avantea, Cremona, Italy

**Keywords:** Neu5Gc, CMAH, αGal, GGTA1, knock out, cattle, pig

## Abstract

The two major sialic acids described in mammalian cells are the N-glycolylneuraminic acid (Neu5Gc) and the N-acetylneuraminic acid (Neu5Ac). Neu5Gc synthesis starts from the N-acetylneuraminic acid (Neu5Ac) precursor modified by an hydroxylic group addition catalyzed by CMP-Neu5Ac hydroxylase enzyme (CMAH). In humans, CMAH was inactivated by a 92 bp deletion occurred 2–3 million years ago. Few other mammals do not synthetize Neu5Gc, however livestock species used for food production and as a source of biological materials for medical applications carry Neu5Gc. Trace amounts of Neu5Gc are up taken through the diet and incorporated into various tissues including epithelia and endothelia cells. Humans carry “natural,” diet-induced Anti-Neu5Gc antibodies and when undertaking medical treatments or receiving transplants or devices that contain animal derived products they can cause immunological reaction affecting pharmacology, immune tolerance, and severe side effect like serum sickness disease (SSD). Neu5Gc null mice have been the main experimental model to study such phenotype. With the recent advances in genome editing, pigs and cattle KO for Neu5Gc have been generated always in association with the αGal KO. These large animals are normal and fertile and provide additional experimental models to study such mutation. Moreover, they will be the base for the development of new therapeutic applications like polyclonal IgG immunotherapy, Bioprosthetic Heart Valves, cells and tissues replacement.

## Introduction

Sialic acids are monosaccharide expressed on the cell surface. They are incorporated as terminal residues in different types of glycoproteins and glycolipids. They are responsible for cell-to-cell and cell-to-microenvironmental interactions. The two major sialic acids described in mammalian cells are the N-glycolylneuraminic acid (Neu5Gc) and the N-acetylneuraminic acid (Neu5Ac). Neu5Gc synthesis starts from the N-acetylneuraminic acid (Neu5Ac) precursor modified by an hydroxylic group addition catalyzed by CMP-Neu5Ac hydroxylase enzyme (1). In non-human mammals, the CMP-Neu5Ac hydroxylase enzyme is coded by the CMAH gene, that was inactivated in humans by a 92 bp deletion that occurred 2–3 millions years ago (2, 3). Only recently have new studies demonstrated that CMAH inactivation occurred independently in the new world monkeys (4) and in other mammals (5), suggesting that the Neu5Gc loss and production of protective Anti-Neu5Gc antibodies could be explained in different mammals as an evolutionary resistance mechanism to different pathogens (6).

The CMAH gene was firstly knocked out in mice (7) generating several “human-like” phenotypes such as the induction of Anti-Neu5Gc antibodies with a possible deleterious interaction with endothelia and epithelia after nutritional incorporation of Neu5Gc, a trend for increased inflammation and immune responses, enhanced immune clearance of recombinant Neu5Gc therapeutics (8), delayed skin wound healing, age-related hearing loss, sexual selection through Neu5Gc antigenicity (9), altered susceptibility to muscular dystrophy (1, 10). Indeed, although humans cannot synthetize the CMP-Neu5Ac hydroxylase enzyme, traces of Neu5Gc are integrated into the membranes of human epithelial and endothelial cells (EC) and more efficiently in malignant cells via food intake. The red meats (beef, pork, and lamb, particularly in processed forms) and dairy products have high contents of Neu5Gc residues, while they are absent in poultry and fish. However, a simple Neu5Gc rich diet exposure, cannot elicit Anti-Neu5Gc antibodies in CMAH^−/−^ mice. In fact, specific Anti-Neu5Gc antibodies were obtained only following strongly immunogenic agents (such as xenogeneic cells) or when a human-specific commensal/pathogen (as the non-typable Haemophilus influenza = NTHi) expressing some adsorbed Neu5Gc residues were used (11). The low antigenic properties of purified Neu5Gc residues was confirmed by another group (12) and, more recently, by Frei et al. immunizing the Neu5Gc-null mice with non-microbial Neu5Gc (13).

Although the possibility that diet induced Anti-Neu5Gc antibodies could have pathological effects in vascular diseases or oncogenesis is still debated in humans, there is evidence for the role of Anti-Neu5Gc in rejection of animal derived tissues [see Salama et al. for review (14)]. Anti-Neu5Gc are elicited by engineered pig skin (15, 16). Pancreatic islets are rejected by CMAH^−/−^ null recipients mice after allotransplantation (12). However, in contrast to Anti-Gal, the presence of Anti-Neu5G antibodies does not seem to trigger hyperacute vascular rejection of Neu5Gc positive chimpanzee kidney in humans [reviewed in Salama et al. (14)] or in a islets transplantation model in Neu5Gc KO mice recipients (12). (See also the detailed review of the role of the elicited Anti-Neu5Gc antibodies recipients of organ and tissue xenotransplantation by Bach et al. in this journal issue). Anti-Neu5Gc antibodies also contribute to modify the pharmacology of therapeutic animal polyclonal IgG (14). In this last case, the rise of high titers of elicited Anti-Neu5Gc antibodies likely contributes to the serum sickness diseases occurring in almost all non-immunosuppressed patients (17, 18).

Developing large animals lacking the major xeno-antigens has thus become a key issue to provide animal vascularized or engineered xenogeneic tissues or glycosylated molecules lacking major xeno-antigens such as αGal, Neu5Gc, or β4GalNT2 in order to decrease the acute or delayed xenograft rejection mediated by the corresponding antibodies. Furthermore, the absence of Neu5Gc will alleviate the possible deleterious effect of elicited Anti-Neu5Gc antibodies on the xenograft recipients own endothelial cells displaying traces of dietary derived Neu5Gc. Finally, it is possible that some patients with red meat intestinal allergy develop IgE Anti-Neu5Gc antibodies (as for Anti-Gal IgE), suggesting by analogy that meat and milk also lacking Neu5Gc, may alleviate such symptoms. In this article, we review the methodological advances that allowed the engineering of pigs and bovine lacking the Neu5Gc alone or in association to other xeno antigens and their potential future value in human pathology and health.

## Methodological Aspects of Gene CMAH (Cytidine Monophosphate-N-Acetylneuraminic Acid Hydroxylase) Inactivation in Large Animals

The last 10 years have seen the advent and development of programmable nucleases for precise editing of the genome (19–22). What was used in the past to achieve genetic modification essentially was a by-product of the cell DNA repair mechanisms (NHEJ, non-homologous end-joining or HDR, homology directed repair), taking advantage of the double strand DNA breaks occurring spontaneously at a very low pace throughout the genome. Now the frequency of DNA breaks is enhanced by a few logs times by the precise cutting ability of the programmable nucleases at selected target sequences. Amongst the programmable nucleases used today for genome editing (Table 1), CRISPR/Cas9 is the most widely used because of its ease to use, flexibility (23), and low cost. The full exploitation of these technologies requires accurate DNA sequencing data as well as the software tools necessary for nuclease design, target site selection, and experimental validation (24–26) and to avoid undesired side effects in other genomic loci. Moreover, compared to the previous techniques of gene targeting, nuclease-based approach does not require the insertion of foreign DNA in the form of selectable markers. These types of nucleases have all been used to successfully edit the genome in a variety of organisms including livestock species both for agricultural (27, 28) and biomedical applications (29, 30), to mention only a few. In the biomedical arena, one species of long standing interest for genetic modification has been the pig for xenotransplantation research, usually targeting one specific locus (31), or two (32). More recently, CRISPR/Cas9 has been implemented to a degree of efficiency to obtain the multiple simultaneous mutations of 3 xenoantigens in the pig (33, 34), including the CMAH gene responsible for the synthesis of Neu5Gc. Even more impressive has been the work reported by Niu et al. (35) with the simultaneous inactivation of all copies of PERVs in the porcine genome. The KO of the enzyme CMAH (cytidine monophosphate-N-acetylneuraminic acid hydroxylase) responsible for the Neu5Gc antigen in pigs has been achieved by several groups, always in association with the simultaneous targeting of the α(1,3) galactosyltransferase (encoded by the GGTA1 gene), using Zn-finger nucleases (32, 36), with TALENs (37), with CRISPR/Cas9 (34), and more recently by the KO of β4GalNT2 (β1,4-N-acetylgalactosaminyltransferase) gene, done simultaneously with the KO of GGTA1 and CMAH (33), again with CRISPR/Cas9. However, there is no public information on breeding successive generation of these pigs, nor is it available on pigs KO only for Neu5Gc. We have generated such animals by breeding αGal and Neu5GC KO male founders (DKO) to wild type sows. Since these mutations are transmitted in a Mendelian fashion, we were able to select in the F2 generation animals KO only for CMAH at the expected frequency (our unpublished observations). Neu5Gc null females were bred to DKO boars and reproduced normally, therefore although there has not been a systematic evaluation of significant numbers, there is no evidence that Neu5Gc KO pigs have major health or welfare issues, nor issues regarding growth rate or breeding ability. Since abnormal islets have been claimed to be associated to the CMAH^−/−^ phenotypes (38), we further assessed the morphology and functionality of pancreatic islets from DKO pig compared to wild type counterparts (39). *In vitro* DKO pig islets exhibited normal insulin secretion after stimulation, there were also no islet histological abnormalities suggesting that the background of KO mice could have been responsible for such phenotype.

**Table 1:** Comparison of different programmable nuclease platforms used in livestock genome editing [adapted from Cox et al. (22) with permission from the Publisher].

	**Zinc finger nuclease**	**TALEN**	**Cas9**
Recognition site	Typically 9–18 bp per ZFN monomer, 18–36 bp per ZFN pair	Typically 14–20 bp per TALEN monomer, 28–40 bp per TALEN pair	22 bp [20-bp guide sequence + 2-bp protospacer adjacent motif (PAM) for *Streptococcus pyogenes* Cas9]; up to 44 bp for double nicking
Specificity	Small number of positional mismatches tolerated	Small number of positional mismatches tolerated	Positional and multiple consecutive mismatches tolerated
Targeting constraints	Difficult to target non-G-rich sequences	Five targeted base must be a T for each TALEN monomer	Targeted sequence must precede a PAM
Ease of engineering	Difficult; may require substantial protein engineering	Moderate; requires complex molecular cloning methods	Easily re-targeted using standard cloning procedures and oligo synthesis
Immunogenicity	Likely low, as zinc fingers are based on human protein scaffold; FokI is derived from bacteria and may be immunogenic	Unknown; protein derived from *Xanthamonas* sp.	Unknown; protein derived from various bacterial species
Ease of *ex vivo* delivery	Relatively easy through methods such as electroporation and viral transduction	Relatively easy through methods such as electroporation and viral transduction	Relatively easy through methods such as electroporation and viral transduction
Ease of *in vivo* delivery	Relatively easy as small size of ZFN expression cassettes allows use in a variety of viral vectors	Difficult due to the large size of each TALEN and repetitive nature of DNA encoding TALENs, leading to unwanted recombination events when packaged into lentiviral vectors	Moderate: the commonly used Cas9 from *S. pyogenes* is large and may impose packaging problems for viral vectors such as AAV, but smaller orthologs exist
Ease of multiplexing	Low	Low	High

**Figure 1:**
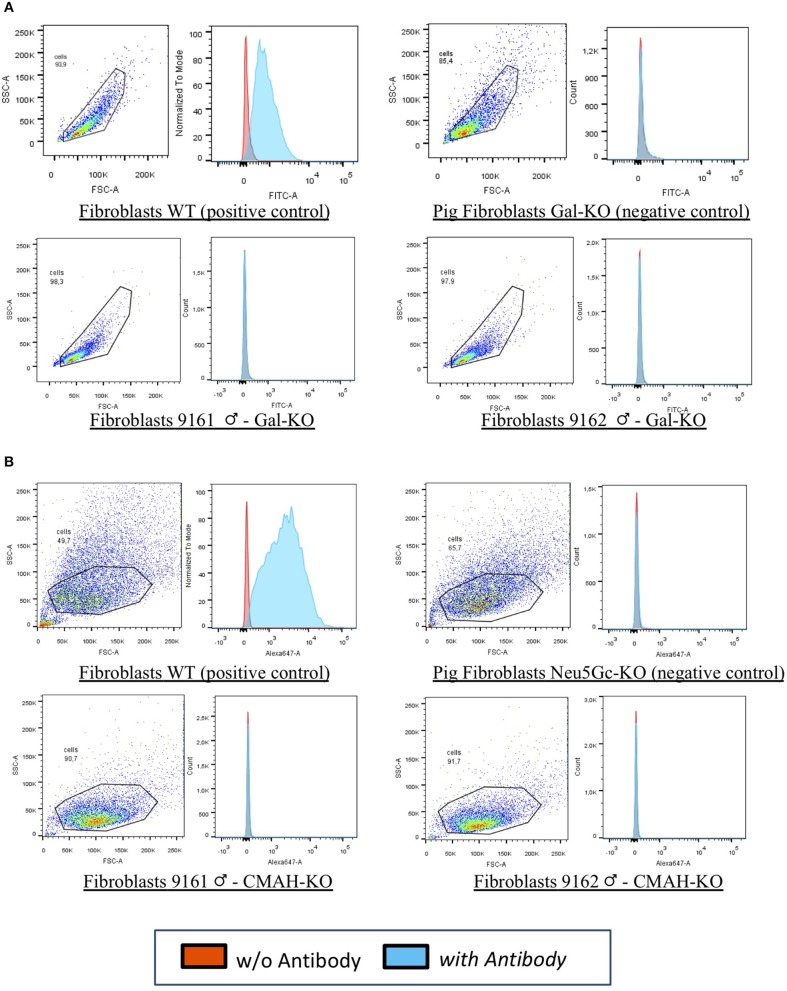
FACS analyses for 9161 and 9162. Fibroblasts from wild type animal (WT) and from the edited calves 9161 and 9162 were analyzed by FACS. As negative controls pig DKO fibroblasts were used as no bovine material was available. The results demonstrated that the αGal **(A,B)** Neu5Gc antigens were absent from the cell surface of cloned calves, confirming the genotype analyses for the knocked-out genes (GGTA1 and CMAH). **(A)** Fibroblasts WT (positive control): wild type primary fibroblasts from the bovine line prior to genetic modification expressing the αGal and the Neu5Gc antigens. Pig Fibroblasts Gal-KO and Neu5Gc-KO (negative control): porcine primary fibroblasts NOT expressing the αGal and the Neu5Gc antigens. Fibroblasts 9161/9162 Gal-KO and Neu5Gc-KO: primary fibroblasts derived from cloned DKO calves [from Perota et al. (40) with permission of the publisher].

In our laboratories, we have recently generated the first cattle line knock out for both αGal and Neu5Gc using CRISPR/Cas9 technology and immunobeads selection (40). We have selected bovine fibroblasts carrying the bi-allelic inactivation of two enzymes including α(1,3) galactosyltransferase (encoded by the GGTA1 gene) and CMP-Neu5Gc hydroxylase (encoded by the CMAH gene) that are not functional in humans. Then, using somatic cell nuclear transfer (41) we generated live calves that do not express the two antigens (Figure 1). Because of the long generation interval in cattle compared to the pig, we have successfully edited both males and females founders. One male founder has reached puberty and semen was collected and cryopreserved for breeding purposes. Ejaculation parameters are normal and when the semen was used for *in vitro* fertilization, blastocyst stage embryos were obtained (our unpublished observations) at the same rate of WT bulls, demonstrating its fertility.

### Neu5Gc-Null Large Animals in Applications and Diseases

The potential value of the Neu5Gc KO in biomedical or biotechnology applications is currently investigated in KO mice (42) or on *in vitro* models (43), given that large animal models KO for Neu5Gc have only recently become available. Clinical and experimental evidences demonstrated that when animal derived biologicals including cells, tissues or molecules such as Ig, are implanted or administered to patients, there is a strong Anti-Neu5Gc immune response, detectable years after the immune challenge such as with non-decellularized pig skin (16). High titers of Anti-Neu5Gc antibodies are also observed in patients receiving rabbit polyclonal IgG, all of them presenting a serum sickness disease (SSD) in the absence of immunosuppressants (IS) (18). The possibility that elicited Anti-Neu5Gc may induce vascular complication has been recently addressed in humans using experimental conditions closer to physiological ones with immune affinity purified diet derived and elicited Anti-Neu5Gc, and endothelial cells loaded with physiological amount of Neu5Gc (44). These data do not suggest that elicited (or diet derived) Anti-Neu5Gc induce an inflammation of endothelial cells but rather suggest that Anti-Neu5Gc may contribute to the homeostasis of ECs. These data are in agreement with the fact that all humans harbor substantial level of Anti-Neu5Gc which has not been eliminated by evolution. Why elicited Anti-Neu5Gc Abs do not result in a clear inflammatory profiling of the EC transcriptome remains unknown. Circulating Anti-Neu5Gc Abs show extremely large differential reactivities with multiple Neu5Gc-containing glycans in an array format. Such a huge epitope conformational diversity may result in a low concentration of Abs for a given EC surface membrane target. Such a situation combined with the yet unknown mechanisms shaped by evolution to cope with the condition resulting from the concomitant presence of natural Anti-Neu5Gc Abs and their target on EC may ultimately result in signals that remain below the activation threshold of ECs. To some extent, Anti-Neu5Gc Abs may thus behave as other “natural antibodies” found in all normal human sera that react against a variety of autologous antigens. Beside the specific safety issue linked to severe SSD, occurrence of a serum sickness disease in patients treated by polyclonal IgGs of animal origin for severe infectious diseases or toxins related in non-immunosuppressed patients, it would also add its own symptomatology to the initial diseases for which the polyclonal antibodies were used. The availability of low immunogeneic, animal derived IgG for therapeutic purposes in non-IS patients is therefore a crucial need (45). In immunosuppressed patients, the Anti-Neu5Gc response resists more to immunosuppressive drugs than the Anti-Gal one does (17), it is however conceivable that Ig from Neu5Gc KO donor would substantially decrease the early toxicity of animal polyclonal IgG administered in the absence of IS such as in the case of Anti-toxins or Anti-infectious agents polyclonal preparations (46). Thus, given the important potential of polyclonal IgG therapeutic applications (47) and the high incidence of SSD mentioned in all studies in patients receiving the foreign Ig in the absence of immune suppression, the generation and availability of low immunogeneic animal derived Igs would be crucial. Approaches to produce human polyclonal antibodies in cattle and to decrease the protein backbone antigenicity included transchromosomic calves KO for the bovine immunoglobulin genes but carrying, on an artificial chromosome, the sequence of the human ones. However, in order to have an efficient immunoglobulin production in these animals, the authors had to “bovinize” the human genes by replacing the constant domain of the human genes (48, 49) with that of cattle creating chimeric immunoglobulins. However, because of the presence of the bovine backbone these antibodies still carry Neu5Gc and therefore will be highly immunogenic. The availability of cattle KO for Neu5Gc will solve this issue and may provide more safety and efficacy to polyclonal antisera immunotherapy, not only in cattle but also in other species used for these purposes like horse, pig, goat and sheep. For the same purpose and more specifically for bioprosthetic heart valves (BHV) manufacturing (seventy per cent of the BHV currently used in the clinic, are in fact manufactured with bovine pericardia), we have generated a cattle line knock out for both αGal and Neu5Gc (40). Although there are reports indicating that the current protocols for processing tissues for BHV manufacturing does not eliminate αGal (50) or Neu5Gc (51) there is yet no published report on the possible Anti-Neu5Gc antibodies response against pig or bovine Bioprosthetic Heart Valves (BHV), Anti-Neu5Gc antibodies may play a role in SVD (Structural Valve Degeneration), preventing the use of BHV in young patients (52, 53). BHV are routinely used in aortic valve replacements but, even if they are made using glutaraldehyde fixed porcine or bovine tissues (valves or pericardium), glutaraldehyde treatment is not sufficient to remove all the donors' glycans xenoantigens. Detection of αGal antigens (50) and of Neu5Gc residues (51), on BHV currently on the market, has been clearly demonstrated, suggesting by analogy a possible role for an immune based mechanism contributing to the SVD of BHV (54–57). To the best of our knowledge there is no study with sufficient statistic power demonstrating the association of BHV elicited anti-Neu5Gc and SVD. Of note the European sponsored FP7 Translink program is currently gathering the data obtained on a large multicentre cohort of BHV recipients where Anti-Neu5Gc levels and repertoire (as well as Anti-Gal) have been measured. Data from this study are expected for early 2020.

### Neu5Gc-Null Large Animals in Food and Health

A largely unexplored avenue is the relevance of Neu5Gc intake through the diet of livestock derived products, both red meat and dairy products, especially for their high Neu5Gc content. As discussed above, diet is the source of a low level Neu5Gc and its accumulation in epithelial and endothelial cells where it could become the target of Anti-Neu5Gc antibodies. The level of such Anti-Neu5Gc antibodies is variable amongst individuals (44, 58–60) and it is not known if the presence of these “xeno auto antigens” is deleterious, as hypothesized (61). If Neu5Gc is incorporated into human tissue through the diet, at least in some individuals then the consumption of red meat or dairy products coming from KO animals should avoid the accumulation into tissues and reduce this risk factor. If this hypothesis will be further validated with additional evidence, then Neu5Gc KO animals could find their way into the food chain for some categories of individuals or infants at risk for specific diseases. Delayed allergic reaction following red meat consumption in some individuals, previously sensitized to αGal following a tick bite and exhibiting a stress-induced T helper-2 (Th2) shift favoring the production of IgE Anti-Gal, is an emergent problem. This allergic reaction, defined as read meat syndrome, has been shown to be related to a type 2 phenotype shift with the production of IgE Anti-Gal (62, 63), may also happen if IgE Anti-Neu5Gc are produced. For these reasons, molecules that are absent in humans but present in large animals that are used as a potential source of bio-products, have been knock out using genetic engineering techniques combined with Somatic Cell Nuclear Transfer (SCNT, animal cloning). αGal has been knock out in the pig by several research groups (64, 65) and this genetic background has become the basis on which to build further genetic modifications for xenotransplantation research. Because of its absence in humans, and its presence in most mammals, Neu5Gc was the second target for genetic engineering for its high immunogenicity in humans as described above.

## Conclusions

Contrary to the αGal antigens, that are immediately degraded, the Neu5Gc antigens from diet are integrated daily by the human cells in their carbohydrates, becoming a constant potential xeno-autoantigen in humans. The continuous exposure to Neu5Gc residues, present in the diet through products of animal origin or used as vital supports in various medical treatments, may theoretically result in a state of persistent inflammation tentatively called “xenosialitis.” This condition has been proposed as an explanation of various diseases that characterize our species, despite the elimination of the Neu5Gc antigen and the development of Anti-Neu5Gc antibodies in humans having been an evolutionarily advantage in resisting to pathogens (66). Further studies are needed to assess the eventual susceptibility of Pigs and Cattle lacking Neu5Gc to human pathogens like for example Influenza A Virus (IAV). It is known for many years that the ferret is the best model for IAV infection studies and it has been demonstrated that the ferret lacks Neu5Gc (67). The availability of Neu5Gc KO livestock (cattle and pigs) that are our main source of animal protein can offer innovative solutions and opportunities in various fields. As it has been the case for the mouse, the first KO model available, the effect of such mutations on the pathophysiological state of animal models closer to humans can be studied in more details. A further possibility, offered by large animal KO models, is to use them as an “humanized” model in xenotransplantation research or diseases modeling. With such KO pigs and cattle it is now possible to source bioproducts for use in medical treatments from less immunogenic sources with great potential benefits (safety and efficacy) to the patients. Last but not least, if the dietary accumulation of Neu5Gc turns out to be a risk factor for some category of human patients, then there is a scope to breed such Neu5Gc-null livestock for food production as well.

## Data Availability Statement

The raw data supporting the conclusions of this manuscript will be made available by the authors, without undue reservation, to any qualified researcher.

## Author Contributions

AP and CG reviewed the literature and wrote the paper.

### Conflict of Interest

AP and CG are employed by Avantea.
